# Natural compounds target programmed cell death (PCD) signaling mechanism to treat ulcerative colitis: a review

**DOI:** 10.3389/fphar.2024.1333657

**Published:** 2024-02-09

**Authors:** Bo Chen, Xinqian Dong, Jin Long Zhang, Xitong Sun, Lin Zhou, Kangning Zhao, Hualiang Deng, Zhen Sun

**Affiliations:** ^1^ Shandong University of Traditional Chinese Medicine, Jinan, China; ^2^ Affiliated Hospital of Shandong University of Traditional Chinese Medicine, Jinan, China

**Keywords:** uc, natural product, inflammation, mechanism, traditional Chinese medicine, PCD

## Abstract

Ulcerative colitis (UC) is a nonspecific inflammatory bowel disease characterized by abdominal pain, bloody diarrhea, weight loss, and colon shortening. However, UC is difficult to cure due to its high drug resistance rate and easy recurrence. Moreover, long-term inflammation and increased disease severity can lead to the development of colon cancer in some patients. Programmed cell death (PCD) is a gene-regulated cell death process that includes apoptosis, autophagy, necroptosis, ferroptosis, and pyroptosis. PCD plays a crucial role in maintaining body homeostasis and the development of organs and tissues. Abnormal PCD signaling is observed in the pathological process of UC, such as activating the apoptosis signaling pathway to promote the progression of UC. Targeting PCD may be a therapeutic strategy, and natural compounds have shown great potential in modulating key targets of PCD to treat UC. For instance, baicalin can regulate cell apoptosis to alleviate inflammatory infiltration and pathological damage. This review focuses on the specific expression of PCD and its interaction with multiple signaling pathways, such as NF-κB, Nrf2, MAPK, JAK/STAT, PI3K/AKT, NLRP3, GPX4, Bcl-2, etc., to elucidate the role of natural compounds in targeting PCD for the treatment of UC. This review used (ulcerative colitis) (programmed cell death) and (natural products) as keywords to search the related studies in PubMed and the Web of Science, and CNKI database of the past 10 years. This work retrieved 72 studies (65 from the past 5 years and 7 from the past 10 years), which aims to provide new treatment strategies for UC patients and serves as a foundation for the development of new drugs.

## 1 Introduction

**FIGURE 1:**
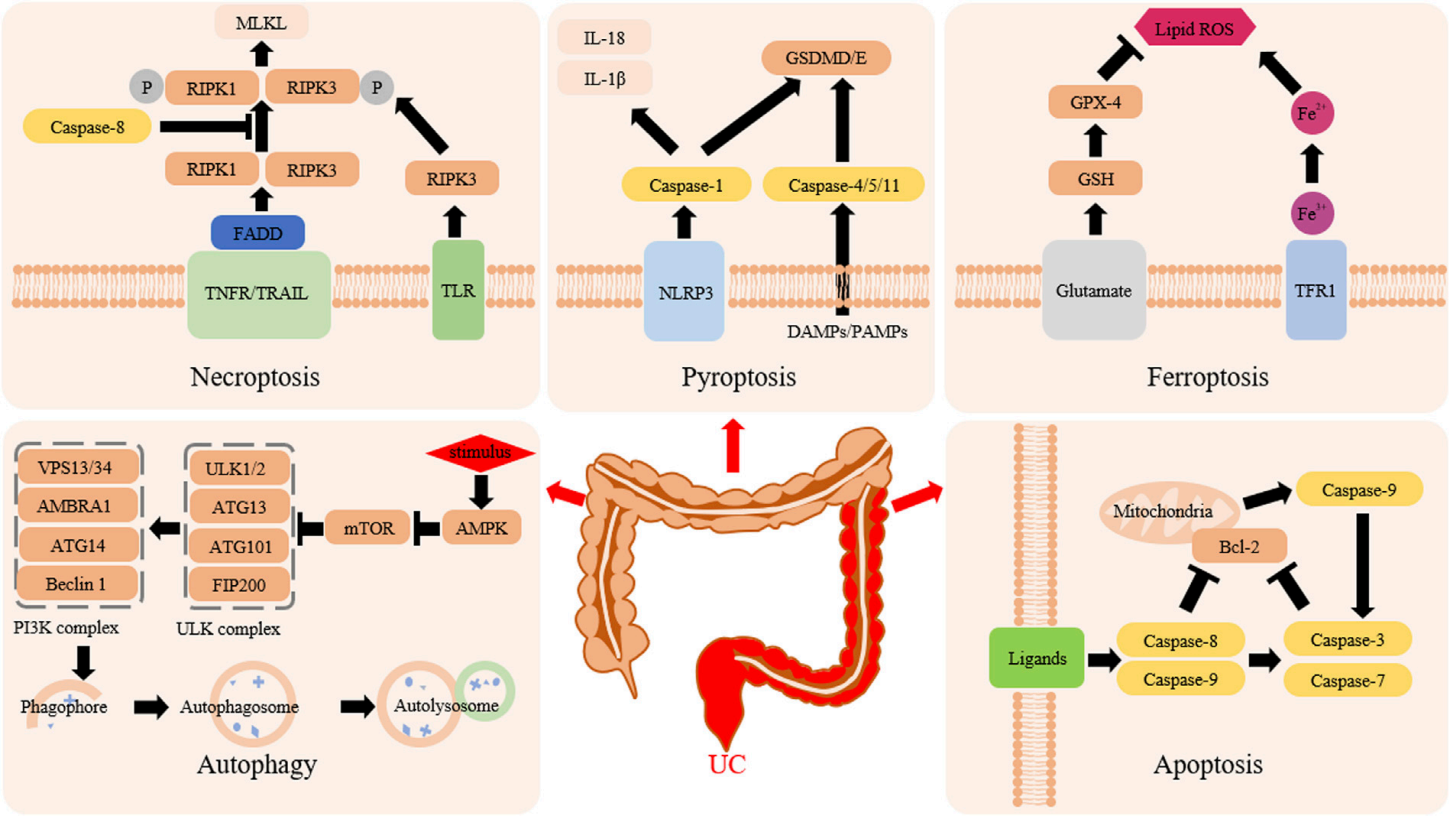
Mechanism of PCD in UC.

Ulcerative colitis (UC) is an inflammatory disease that begins in the rectum and involves the entire intestinal mucosa continuously. The main clinical symptoms are abdominal pain, mucus, pus and bloody stools, and tenesmus. It was reported that the global prevalence of UC would be approximately 5 million cases in 2023, and the incidence rate is still increasing worldwide (Le Berre et al., 2023), which undoubtedly imposes a huge burden on the global medical and economy. Currently, corticosteroids, aminosalicylic acid preparations, and some biological agents are used in the clinical treatment of UC. Still, adverse events such as high drug resistance rates, complications, and risk of tumor formation are worrying (Eisenstein M., 2018). Therefore, seeking new treatment strategies has become an urgent problem that needs to be solved now. Programmed cell death (PCD) is a death method that involves complex signals, occurs in an orderly manner, and is regulated by genes. It mainly includes apoptosis, autophagy, ferroptosis, pyroptosis, and necroptosis (Figure 1). PCD is activated and executed by the cells themselves. It plays an important role in the normal development of multicellular biological tissues, maintaining homeostasis of the home environment and clearing damaged cells (Bedoui et al., 2020). A large amount of evidence shows PCD is abnormally expressed in the occurrence and development of UC, which means that targeted regulation of PCD may be a new strategy for the treatment of UC (Li et al., 2022). More experiments have proven that targeted regulation of different molecular mechanisms and targets in other forms of PCD can significantly reduce the inflammatory response of UC and alleviate pathological damage, colon shortening, and weight loss (Wan et al., 2022).

**FIGURE 2:**
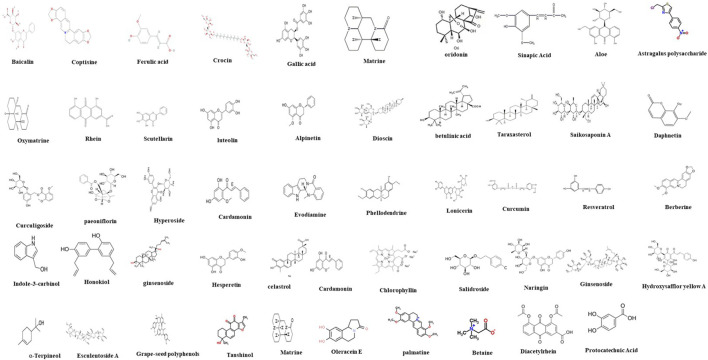
Chemical structures of natural compounds.

In recent years, natural compounds have been widely used in a variety of diseases, such as hypertension, diabetes, liver cancer, etc., and have attracted much attention due to their huge therapeutic potential (Piragine et al., 2022; Zhu et al., 2017; Anwanwan et al., 2020). Excitingly, some natural products have emerged as candidates for the treatment of UC, and these compounds have been shown to protect the colon and reduce inflammation by modulating different types of PCD. Therefore, these natural compounds can be complementary and alternative treatments for UC (Figure 2).

## 2 The mechanism of action of PCD in UC

### 2.1 Apoptosis

Apoptosis is a PCD, which plays an important role in the growth and development of the body and the homeostasis of tissues and organs. Under certain physiological or pathological conditions, cells can undergo morphological changes such as shrinkage, chromatin condensation, and apoptotic body formation (Xu et al., 2019). More and more studies have shown that targeted induction of apoptosis can effectively antagonize tumor progression and reduce resistance to chemotherapy drugs (Kulbay et al., 2022). The process of apoptosis involves three stages: 1) Pre-apoptosis: the cell membrane conformation and permeability change, and the initial Caspase protein is activated. 2) Middle stage of apoptosis: the effector Caspase protein is activated, and cleavage is completed. 3) Later stage of apoptosis: DNA fragmentation, formation of apoptotic bodies, and phagocytosis by phagocytes (Carneiro and El-Deiry, 2020). Apoptosis exerts biological effects in cells mainly through the intrinsic pathway initiated by mitochondria and the exogenous pathway initiated by death receptors. As for the exogenous pathway, death ligands (TNF family members such as Fasl) bind to death receptors (DR), causing death receptors (such as Fas) to aggregate. The aggregated Fas passes through a special death structure in its cytoplasmic region. The death domain (DD) recruit’s adapter protein (FADD) and Caspase-8 to form the death-inducing signaling complex (DISC). DISC activates Caspase-8, and the activated Caspase-8 triggers subsequent Caspase cascade reactions to promote cell death (DArcy., 2019). The occurrence of the intrinsic pathway is usually triggered by intrinsic factors such as hypoxia, metabolic stress, or the inability of damaged DNA to repair itself. When cells are stimulated by apoptotic signals, the pro-apoptotic proteins Bax and Bak induce changes in the permeability of the outer mitochondrial membrane, leading to the release of cytochrome C within the mitochondria. Then, free cytochrome C binds to the apoptotic body Apaf-1 to form a complex, which activates Caspase-9. The activated Caspase-9 protein further activates the effector protein Caspase-3/7 proteins to promote downstream-related signals and cause cell apoptosis (Lin et al., 2022).

In the development of UC, multiple apoptotic signaling molecules are involved in regulating the process. The nuclear factor-kB (NF-κB) family is composed of five members, each subunit containing a Rel homology domain (RHD), which promotes dimerization and DNA binding and undergoes biological effects such as immune defense, apoptosis, proliferation, as well as cell migration and invasion, by either classical or non-classical pathways in cells (Wang et al., 2022; Wang and Shen, 2022). Studies have shown that the overactivation of NF-κB plays a vital role in the occurrence and development of UC (Xiong et al., 2022). Arab et al. confirmed through experiments on TNBS-induced UC rats that inhibiting the NF-κB pathway can inhibit cell apoptosis, reduce inflammatory infiltration, and effectively alleviate colonic inflammatory damage (Arab et al., 2021). Bcl-2 was first identified in B-cell lymphoma, which acts as a repressor gene that plays a vital role in apoptosis. Giriş et al. used Western blotting assay in UC model rats and showed increased expression of Bax and decreased expression of Bcl-2 in this model (Giriş et al., 2008). The JAK/STAT signaling pathway consists of ligand-receptor complex, JAK, and STAT, and is involved in various biological effects such as immune adaptation, tissue repair, inflammation, and apoptosis. It has been reported that the overexpression of inflammatory factors in UC disease aggravates macrophage and neutrophil infiltration, which may be achieved by promoting apoptosis through the JAK/STAT pathway (Caiazzo et al., 2023). Therefore, the degree of pathological damage to intestinal mucosa caused by inflammation can be reduced by inhibiting JAK/STAT. It is not difficult to find that the pathogenic mechanism of UC is complex. In addition to the signaling pathways listed above, it also involves some other pathogenic mechanisms, such as extracellular signal-regulated kinase (ERK), PI3K/AKT/mTOR signaling pathways, etc. The above research results show that the occurrence and development of UC are closely related to apoptosis and are involved through multiple signaling pathways and targets.

### 2.2 Autophagy

Autophagy is a highly conserved process in eukaryotes that maintains normal cell metabolism and the renewal of some organelles (Glick et al., 2010). Under the regulation of autophagy-related genes (Atgs), lysosomes are used to degrade damaged organelles and macromolecules to prevent damage to their cells. In addition, they can survive in the presence of nutritional deficiencies and respond to harmful stimuli. There are three different types of autophagy: microautophagy, macroautophagic, and chaperone-mediated autophagy. Moreover, macroautophagy, which is the main form of autophagy, is defined as autophagy. Autophagy includes basal autophagy under physiological conditions and induced autophagy under stress conditions (Levine and Kroemer, 2019). And the latter is when the cell receives autophagy-inducing signals, a membrane-like structure similar to liposomes is formed at the cytoplasmic membrane, which continuously expands to become a phagophore, and the phagophore, by extending and expanding, encases the components in the cytoplasm into the membrane, and then forms an airtight globular structure, which is called an autophagosome. Autophagosomes fuse with lysosomes to form autolysosomes, and the materials wrapped in them are catabolized and metabolized by hydrolases in the lysosomes to form small molecule substances (such as amino acids, nucleotides, etc.) reuse (DArcy., 2019; Kocaturk et al., 2019). Therefore, regulating autophagy is of great significance for intervening in disease progression. Excitingly, regulating autophagy-related gene proteins has become a new way to antagonize the progression of UC by maintaining the integrity of the intestinal mucosal barrier and reducing inflammatory damage to the colon mucosa.

During the autophagy process, there are many keys signaling pathways involved, which jointly affect the prognosis and outcome of UC. mTOR is a serine/threonine kinase among members of the P13k-related kinase (PIKK) family. As a core signaling protein in the autophagy response process, mTOR regulates cell growth, proliferation, and energy metabolism (Deleyto-Seldas and Efeyan, 2021). Adenosine 5’-monophosphate-activated protein kinase (AMPK) is an important upstream signaling molecule of mTOR, and the activation of AMPK is positively correlated with cellular energy status. When cells have sufficient energy, mTORC1 inhibits the initiation of autophagy by destroying the binding site between ULK1 and AMPK. In the opposite case, it can also inhibit the phosphorylation of MTORC1 and initiate the expression of autophagy (Kim and Guan, 2015). An experimental study found that regulating AMPK/mTOR can significantly improve inflammation in UC model mice and exert a protective effect on the colon mucosa (Gao et al., 2020). As a homolog of Atg6/VPS30, Beclin-1 is an essential molecule for the formation of autophagosomes and plays an important role in autophagy activity by interacting with multiple Atgs (Kaur and Changotra, 2020). It has been reported that maintaining the homeostasis of Beclin-1 to balance autophagy is a feasible solution for the treatment of UC (Dai et al., 2017). In addition, the activation of NF-κB, NLRP3, etc., is positively correlated with the expression of inflammation. By regulating the expression of autophagy, it inhibits the release of pro-inflammatory factors, thereby repairing the colon mucosa and inhibiting the overexpression of inflammation. Therefore, we can find that these target protein molecules are involved in regulating autophagy in UC cells, becoming a new idea for treating UC.

### 2.3 Ferroptosis

Ferroptosis is a type of programmed cell death that is different from other programmed cell death methods, such as apoptosis, necrosis, and autophagy. Its morphological characteristics are as follows: increased density of mitochondrial double-layer membranes, volume shrinkage, and reduction or disappearance of cristae (Li et al., 2020). The mechanism can be described as follows: the expression of intracellular glutathione (GSH), glutathione peroxidase 4 (GPX4), and glutamate-cystine reverse transporter (System, Xc-) is inhibited; at the same time, lipid peroxides cannot be cleared in time, resulting in excessive accumulation of reactive oxygen species (ROS) and Fe^2+^ in the body to activate the Fenton reaction, leading to the occurrence of Ferroptosis (Liu et al., 2022). Extensive research has shown that ferroptosis plays a key role in various diseases. Activating or blocking target proteins of it signaling pathway can effectively alleviate the progression of the disease, which will provide alternative treatments for many diseases (Tang et al., 2021). Studies have shown that the expression of the ferroptosis signaling pathway is often accompanied by the participation of several key protein signaling molecules. NFE2-related factor 2 (Nrf2) is a key regulator in the antioxidant response and can participate in various downstream target gene interventions or correct redox imbalance (He et al., 2020). It is worth noting that the Nrf2 target gene can not only regulate key processes of iron metabolism but also regulate the catabolism of xenobiotics and reactive aldehydes, as well as GSH synthesis and NADPH regeneration, making Nrf2 a multifaceted regulator of anti-iron degradation reactions (Dodson et al., 2019). GPX4 is a member of the selenium-containing family with a molecular weight of about 19 kDa and is composed of 170 amino acids. It can use its antioxidant activity to inhibit the overexpression of lipid peroxides and convert lipid hydroperoxides into lipids. It can improve the quality of lipocalciferol and prevent iron (Fe^2+^)-dependent formation of toxic lipid ROS, inhibiting the occurrence of ferroptosis events (Zhang et al., 2022). In addition, P53 can also inhibit the normal function of Xc-by regulating cystine levels, thereby affecting GPX4 activity, leading to insufficient antioxidant capacity of the body and inducing ferroptosis (Jiang et al., 2015). Therefore, GPX4 is considered a key regulator in the ferroptosis pathway and can also be used as an important indicator for evaluating ferroptosis. It is reported that ferroptosis-related signaling pathways are important targets for the treatment of UC, and regulating ferroptosis can effectively alleviate the pathological changes of UC and the occurrence of colon cancer. In an experiment, it was found that inhibiting the expression of the Nrf2 signaling pathway can promote the occurrence of ferroptosis. At the same time, more severe colon inflammation and colon cancer risk were observed in UC model mice. Activating this signal can reverse it (Huang et al., 2022). In addition, increasing GPX4 activity can effectively improve colon histological damage and intestinal epithelial barrier dysfunction and reshape intestinal microecology (Wang et al., 2023). In addition, experiments using DSS-induced NCM460 cells revealed that activation of the Nrf2-GPX4 signaling pathway could effectively protect intestinal epithelial cells from Ferroptosis injury and alleviate UC (Dong et al., 2021). The above studies show that ferroptosis has great potential in the treatment of UC, thus opening up new avenues for the future development of drugs related to the treatment of UC.

### 2.4 Pyroptosis

Pyroptosis is a type of cell inflammatory necrosis mediated by the Gasdermin D (GSDMD) protein family. This process is mainly achieved through the classical pathway of Caspase-1 and the non-canonical pathway of Caspase-4/5/11 (Fang et al., 2020). When external stimuli stimulate cells, the cells swell, causing the cells to rupture. Many inflammatory factors, such as IL-1β and IL-18, are released outside the cells through the membrane pores formed by GSDMD, causing a robust inflammatory response and subsequent pyroptosis. Regarding the treatment of UC, pyroptosis is undoubtedly a promising new approach. It can improve the efficiency of treatment and the prognosis of UC, develop new inhibitors, and reduce drug resistance (Zhang et al., 2020). In addition, GSDMD and NLR family pyrin domain-containing protein 3(NLRP3) are important targets and have positive significance in UC disease. Inhibiting the expression of NLRP3 and GSDMD can reduce intestinal immune response, improve pathological tissue structure, and exert intestinal protective effects (Sun et al., 2021; Ye et al., 2023). Therefore, targeting the regulation of pyroptosis-related signaling pathways to treat UC may be an effective strategy.

### 2.5 Necroptosis

Necroptosis is a process that causes abnormal cells to self-destruct. Mechanistically, it does not depend on the activation of Caspase-8 but requires activated receptor-interacting protein kinase 3 (RIPK3) to phosphorylate mixed lineage kinase domain-like protein (MLKL) to form a pore complex on the plasma membrane, which in turn directly activates or regulates the inflammatory response through the released cellular content (Khoury et al., 2020). It is worth noting that Günther et al. reported that protein factors such as receptor-interacting protein kinase 1 (RIPK1), RIPK3, and MLKL have potential positive effects in the treatment of UC (Günther et al., 2013). Similarly, Lee *et al.* used DSS to establish UC model mice and observed significant increases in IL-17, IL-6, TNF-α, RIPK3, and MLKL expression levels. Treatment with RIPK3 inhibitors alleviated these changes, improving intestinal inflammation and structural damage (Lee et al., 2020). The same study found that RIKP1 inhibitors can prevent sustained damage to intestinal tissue caused by inflammatory factors such as TNF-α, IL-1β, IL-22, and IL-6 mRNA while inhibiting cell necroptosis. This may offer a therapeutic option and new hope for UC (Zeng et al., 2022). Additionally, RIPK3 deficiency can alleviate the production of inflammatory factors (IL-16, IL-17, and IFN-γ) and ROS in UC mice, providing potential evidence for the use of RIPK3 inhibitors in treating UC disease (Duan et al., 2022). These studies demonstrate that RIPK1/RIPK3/MLKL-regulated necroptosis can protect colon tissue from inflammation and preserve the intestinal mucosal barrier function.

## 3 Compounds prevent UC by regulating cell death

### 3.1 Compounds that regulate the apoptotic pathway

A large amount of clinical data has proven that natural compounds can improve the pathological tissue state of UC by inhibiting the apoptosis pathway, downregulating the expression of inflammation, and restoring colonic mucosal homeostasis. This therapeutic pathway is related to NF-KB, Bcl-2, JAK/STAT, ERK, ER, PI3K/AKT/mTOR, and oxidative stress. This will provide a reliable theoretical basis for the treatment of UC with natural compounds.

#### 3.1.1 Compounds related to the NF-κB signaling pathway

NF-κB is a key transcriptional regulator that affects disease outcome or prognosis through classical or non-classical pathways. It can not only promote the expression of inflammatory factors but also regulate the differentiation and apoptosis of immune cells. Therefore, using natural compounds to interfere with NF-κB has become a potential means of treating diseases. It is reported that some natural compounds can regulate NF-κB to inhibit apoptosis and can effectively curb the progression of UC. For example, baicalin is a flavonoid derived from the dried roots of the Lamiaceae plant *Scutellaria baicalensis* Georgi. Modern pharmacological research has proven that this compound has various pharmacological effects such as anti-inflammation, antioxidant, anti-apoptosis, immune regulation, and maintenance of the intestinal barrier (Wang et al., 2018). Shen et al. investigated the mechanism of action of baicalin on TNBS-induced UC rats, which were randomly divided into five groups: blank, model, and baicalin low, medium, and high dose (30,60,90 mg/kg) groups, and were given gavage treatment to UC rats for 28 days. Compared with the control group, baicalin inhibited the expression of Caspase-3, Caspase-9, Bcl-2/Bax, IL-1β, and TNF-α. It ameliorated the pathological damage of inflammation to the colonic mucosa and the degree of colonic shortening, and the therapeutic effect was dose-dependent. Moreover, it was further verified with the LPS-induced RAW264.7 cell model, which showed that the anti-inflammatory effect of baicalin was exerted by inhibiting the NF-κB signaling pathway. However, unfortunately, the comparison of concentration gradients and the setting of a positive control group were not performed in this experiment, which lacked a certain degree of credibility. Ferulic acid (FA) is a phenolic compound in plant medicines such as *Angelica sinensis* (Oliv.) Diels, and Rhizoma Chuanxiong, which exhibit antioxidant and anti-inflammatory properties (Li et al., 2021). Ghasemi *et al.* treated acetic acid-induced UC model rats for 7 days by using different concentrations (20,40,60 mg/kg) of ferulic acid to observe the inflammation of the colonic mucosa and pathologic damage of the treatment. The results showed that ferulic acid was able to inhibit the expression of mRNA of colon inflammation and apoptosis genes and at the same time, upregulated the antioxidant capacity of CAT and SOD, which effectively protected the integrity of the mucosa of colon tissue. The mechanism was related to the inhibition of LPS-TLR4-NF-κB and NF-κB-INOS-NO signaling pathways (Ghasemi-Dehnoo et al., 2023). Coptisine (COP) is an isoquinoline alkaloid from the dried rhizomes of *Coptis chinensis* Franch. In the buttercup family, with anti-inflammatory, antioxidant, anti-apoptotic, and gastrointestinal protective effects (Wu et al., 2019). Wang *et al.* elucidated the anti-inflammatory mechanism of action of CPO by DSS-induced colitis in mice. The experimental results showed that both COP (25,50,100 mg/kg) and 5-ASA (200 mg/kg) groups were able to effectively alleviate weight loss and Disease Activity Index scores (DAI) and repair intestinal mucosal structure and upregulate the protein expression of ZO-1, ZO-2, occludin, and claudin-1, inhibit TNF-α, IFN-γ, IL -1β, IL-6 and IL-17 inflammation levels and the expression of Bax and Caspase-3. This change was dose-dependent and therapeutically superior to the 5-ASA group. However, no pharmacological toxicity and clinically relevant experimental studies were conducted in this experiment, and the safety and clinical therapeutic efficacy are of concern (Wang et al., 2021). Another study found that oridonin (Ori) (2.10 mg/kg) was used to treat DSS-induced UC model mice for 14 days. Compared with the model group, mice in the high-dose group (10 mg/kg) showed a significant decrease in DAI and a significant increase in colon length. HE showed that Ori reversed the protein levels of ZO-1, occludin, and claudin-1, and ELISA assay revealed a significant downregulation of inflammatory levels of TNF-α, IL-1β, and IL-6 compared with that of the DSS group, and the therapeutic effect of the high-dose group was more prominent. It also enhanced the antioxidant capacity of GSH-Px and SOD and the content of the anti-apoptotic protein Bcl-2. It inhibited the expression of Bax and Caspasee-3, and its mechanism of action is to antagonize the apoptotic pathway by inhibiting the NF-κB signaling pathway and thus exerting an anti-inflammatory and antioxidant effect to protect the structural integrity of intestinal mucosa (Wang et al., 2022). Although this experiment demonstrated that Ori could exert anti-inflammatory effect in the treatment of UC, no positive control group was set to further verify the experimental data’s reliability. In addition, we have identified many compounds that inhibit apoptosis (Table 1 for details), which can also play an essential role in treating UC. The above studies have shown that such compounds can inhibit apoptosis in UC cells through NF-κB signaling as a practical pathway.

**TABLE 1:** Natural compounds target apoptosis signaling pathway to treat UC.

Natural products	Dosage	Models	Biological effects	Results	Mechanism	References
Cardamonin	10,30 mg/kg	UC model rats	iNOS↓NF-κB↓TNF-α↓caspase-3↓	Reduce inflammation, oxidative stress, and apoptosis	MPO↓MDA↓COX-2↓	Ali et al. (2017)
Green Alga	10,50,100 mg/kg	UC model rats	IL-4↑Nrf2↑Mn-SOD↑Caspase-3/8/9↓Bcl-2/Bax↓P53↓	Inhibition of apoptosis and maintenance of intestinal epithelial cell homeostasis	NF-κB/SIRT1/Nrf2↓	Ardizzone et al. (2022)
Crocin	10,20,30 mg/kg	UC model rats	Bax↓caspase-3/8/9↓NF-κB↓TNF-α↓IL-1β ↓ IL-6 ↓cAMP↑Bcl-2↑IL-4↑IL -10↑	Inhibition of apoptosis and restoration of normal weight and length of colon and improvement of morphological structure of colon cells	Bcl-2↑	Albalawi et al. (2023)
Scutellarin	20 mg/kg	UC model rats	TNF-α↓MDA↓NO↓IL-6↓SOD↑Bax↓Bcl-2↑	Inhibit oxidative stress and alleviate pathological damage	SOD↑	Aksit et al. (2023)
Oxymatrine	25,50,100 mg/kg	UC model rats	TNF-α↓IL-6↓IL-1β↓IFN-γ↓IL-17A↓	Inhibition of inflammatory responses	PI3K/AKT↓	Chen et al. (2017)
Taraxasterol	20,50,100 mg/kg	UC model rats	p53↓caspase-3↓IL-6 ↓TNF-α↓	Inhibition of inflammatory responses	Bcl-2↑	Che et al. (2019)
	2.5,5,10 μg/mL	HT-29 cells				
Daphnetin	16 mg/kg	UC model rats	TNF-α↓IL-6↓TNF-α↓Bcl-2↑occludin↑MDA↓SOD↑	Inhibit cell apoptosis and alleviate inflammatory damage	JAK2/STAT3↓	Cordes et al. (2020)
Houttuynia	40,80 mg/kg	UC model rats	TNF-α↓IL-1β↓IL-6↓TLR4↓	Restoring intestinal homeostasis and increasing the richness of gut microbiota	NF-κB↓	Cen et al. (2022)
Matrine	0,0.25,0.5,1,2,4 mg/mL	NCM460 Cells	IL-6↓TNF-α↓IL-1β↓IL-2↓caspase-3↓Bcl-2↑	Inhibit the expression of inflammation and reduce the damage of intestinal epithelial cells	JAK2/STAT3↓	Chen et al. (2022)
Rhein	12.5,25,50 mg/kg	UC model rats	TNF-α↓IL-1β↓IL-6↓PI3K↓Akt↓mTOR↓p70S6K1↓	Reverse intestinal flora and reduce pathogenic bacteria	PI3K/AKT↓	Dong et al. (2022)
	12.5,25,50 μM	RAW264.7 cells				
Ferulic acid	20.40,60 mg/kg	UC model rats	SOD↑CAT↑; MDA↓NO↓mRNA↓	Inhibits the inflammation and histopathological damage of colonic tissue in colitis rats	LPS-TLR4-NF-κB↓; NF-κB-INOS-NO↓	Ghasemi-Dehnoo et al. (2023)
betulin	8 mg/kg	UC model rats	TNF-α↓IL-1β↓IL-6↓Caspase-3/8↓	Inhibition of inflammatory infiltration and improvement of pathological state	TLR4/NF-κB↓	El-Sherbiny et al. (2021)
Chloroform extract	30,60,90 mg/kg	UC model rats	IL-6 ↓TNF-α↓IL-1β ↓Zonula occlusionn-1↑occludin↑Claudin-2↑	Inhibit apoptosis, reduce inflammation and protect intestinal epithelial cells and intestinal epithelial barrier	Bcl-2/Bax↑	Fu et al. (2022)
	1,10,20,40,60,80, 100,120,140 μg/mL	RAW264.7 cells				
Saikosaponin A	12.5,25,50 mg/kg	UC model rats	IKKα↓IKB↓NF-κB p65↓caspase-3↓Bax↓IL-1β↓IL-6↓TNF-α↓Bcl-2↑	Reduce inflammatory response and repair the integrity of colonic mucosal tissue	IKK/IKB/NF-κB↓	Gao et al. (2022)
Graviola	100 mg/kg	UC model rats	Bax↓caspase-3↓MDA↓NO↓MPO↓	Inhibit cell apoptosis and reduce colon injury	Bcl-2↑	Helal andAbd Elhameed (2021)
Ginsenoside RK2	5,10,20,40 μM	Caco-2 cell/THP-1 cell	IL-1β↓TNF-α↓IL-6↓	Inhibit inflammatory response and restore intestinal epithelial function	ERK/MEK↓	Huang et al. (2022)
Oleracein E	5,10,20 mg/kg	UC model rats	SOD↑Bax↓Bcl-2↑caspase-3↓IL-1β↓IL-6↓	Improve intestinal barrier damage and reduce inflammation and oxidative stress	Nrf2/HO-1↑	Huang et al. (2023)
Fermented Glutinous Rice	100,300 mg/kg	UC model rats	Zonula occlusionn-1↑occludin↑Claudin-2↑ IL-6 ↓TNF-α↓IL-1β ↓Caspase-3↓Bax/Bcl-2↓	Inhibit inflammatory response and alleviate UC.	Bcl-2↑	Kim et al. (2023)
α-Terpineol	50 mg/kg	UC model rats	Caspase-3↓Caspase-9↓NF-kB-p65↓Bcl-2↑	Inhibits the inflammation and histopathological damage of colonic tissue in colitis rats	NF-κB↓	Khan et al. (2023)
Esculentoside A	0,4.5,9,18 μM	SMCs cells	IL-6↓TNF-α↓Bax↓Bcl-2↑	Inhibit inflammatory response and alleviate pathological damage	NF-κB↓	Liu et al. (2022)
	20 mg/kg	UC model rats				
Baicalin	30,60,90 mg/kg	UC model rats	SOD↑CAT↑GSH-Px↑; MDA↓IL-Iβ↓TNF-α↓Caspase-3↓Caspase-9↓Bcl-2/Bax↓	Inhibition of apoptosis signaling pathway can reduce the pathological damage of colonic mucosa caused by inflammation	NF-κB↓	Shen et al. (2019)
Aloe	18,72 mg/kg	UC model rats	TNF-α↓IL-1β↓IL-6↓	Enhancing colonic mucus barrier to improve UC	PKC/ERK↓	Shi et al. (2021)
	0,10,20,40 μM	LS174T cells				
Sinapic Acid	40 mg/kg	UC model rats	MDA↓NO↓TNF-α↓IL-6↓Bax↓caspase-3↓	Inhibition of inflammation, oxidative stress and apoptosis in colon tissue significantly improved UC.	Bcl-2↑	Shahid et al. (2022)
Grape-seed polyphenols	500,750 mg/kg	UC model rats	TNF-α↓IL-6↓IL-1β↓mRNA↓	Inhibit the inflammatory response and repair the damaged mucosa	STAT3↓	Wang et al. (2020)
Coptisine	25,50,100 mg/kg	UC model rats	TNF-α↓IFN-γ↓IL-1β↓IL-6↓IL-17↓IκB↓	Inhibits cell apoptosis, protects the integrity of intestinal barrier and alleviates inflammatory cell infiltration	NF-κB p65↓	Wang et al. (2021)
oridonin	2.10 mg/kg	UC model rats	TNF-α↓IL-1β↓ZO-1↑occludin↑claudin-1↑	Inhibition of oxidative stress and apoptosis of intestinal mucosal cells can improve the pathological damage of colon tissue	NF-κB/p53↓	Wang et al. (2022)
Honey Polyphenols	10.5 mg/kg	UC model rats	IL-1β↓IL-6↓TGF-β1↓TNF-α↓ IFN-γ↓GSH-Px↑NO↓	Improve intestinal inflammation and oxidative stress resistance	SOD↑	Zhao et al. (2019)
Gallic acid	20,40,60 mg/kg	UC model rats	IL-1↓IL-6↓IL-12↓IL-17↓IL-23↓TGF-β↓TNF-α↓	Inhibition of inflammatory response and improvement of pathological state	NF-κB↓	Zhu et al. (2019)
Tanshinol	15,30 mg/kg	UC model rats	TNF-α↓IL-6↓IL-8↓IL-1β↓caspase-3↓caspase-9↓Bax↓Bcl-2↑	Reduce inflammatory response and improve cell viability	BCL2↑	Zhu et al. (2021)
	25,50,100 μmol/L	FHC cells				
Piper wallichii	20,50,100 mg/kg	UC model rats	TNF-α↓IL-1β↓IL-6↓occludin↑TLR4↓p-IκB-α↓P65↓COX-2↓	Inhibit cell apoptosis and inflammatory response and improve intestinal barrier function	TLR4/NF-κB/COX-2↓	Zhao et al. (2023)
	10,20,40,80 μg/mL	RAW264.7 cells				

#### 3.1.2 Compounds related to Bcl-2 signaling pathway

The Bcl-2 family consists of regulatory proteins that induce pro-apoptosis or inhibit apoptosis and play a central role in the endogenous apoptosis pathway. Excitingly, it is a crucial target for treating many diseases, including UC (Valentin et al., 2018; Sakurai et al., 2014). Some findings suggest that this class of natural compounds can effectively alleviate UC inflammation and maintain intestinal integrity and mucosal barrier function by upregulating the anti-apoptotic protein Bcl-2. Crocin is a carotenoid compound in the active ingredient of the dried stigmas of *Crocus sativus* L., an iris plant. It has various pharmacological effects such as antioxidant, anti-inflammatory, and anti-cancer. Albalawi *et al.* experimentally explored crocin’s therapeutic effect and mechanism in UC rats. Crocin (10,20,30 mg/kg) was administered orally to treat UC rats for 14 days. Using HE, ELISA, and RT-PCR assays, it was found that crocin was able to improve the morphology and structure of the colonic mucosa and restore the length of the colon, inhibit the expression of Caspase-3/8/9, Bax, IL-1β, IL-6, and TNF-α, and upregulate the content of Bcl-2. Since no positive control group was set up in this study, the effect could not be determined (Albalawi et al., 2023). Sinapic Acid (SA) belongs to the group of polyphenolic compounds which are widely found in vegetables, citrus fruits. It has medicinal properties such as anti-inflammatory and antioxidant properties. Shahid et al. used 40 mg/kg SA orally for 7 days to treat AA-induced UC rats. Prednisolone (10 mg/kg) was used as a control group. The results showed that both 40 mg/kg of SA and 10 mg/kg of prednisolone were able to effectively alleviate the necrosis and erosion of the colonic mucosa. In addition, both treatments were able to reduce the levels of inflammatory factors TNF-α and IL-6, inhibit the overexpression of MDA and NO, alleviate oxidative stress damage and reduce the expression of Bax and Caspase-3 apoptotic proteins, and activate the anti-apoptotic protein Bcl-2 pathway to play a role in protecting the structure of intestinal mucosa, and the therapeutic effect of 40 mg/kg SA was better than that of prednisolone. Taraxasterol is a triterpenoid compound found in the dried whole grass of several species of the Asteraceae plant *Taraxacum mongolicum* Hand. Which has anti-inflammatory properties. Che *et al.* treated DSS-induced UC mice orally with different doses (20,50,100 mg/kg) of taraxasterol. The results showed that taraxasterol alleviated the rate of apoptosis and the level of colonic inflammation and pathologic tissue score in a dose-dependent manner. In addition, serum TNF-α, IL-6, and IL-1 expression were inhibited, and Bax, P53, and Caspase-3 expression was decreased. Meanwhile, *in vitro* experiments with the LPS-induced HT-29 cell model proved this idea. There were flaws in the design of this experiment; for example, no positive control group was set up, and the drug was not administered based on body weight (Che et al., 2019). In addition, we have identified Tanshinol (TAN), the primary phenolic compound of *Salvia miltiorrhiza* Bge. In the family Labiatae, which has attracted much attention because of its various pharmacological effects, including antioxidant and anti-inflammatory effects. Zhu *et al.* used TAN (15.30 mg/kg) to intervene in DSS-induced UC mice for 14 days without setting a positive control group. The results showed that TAN was able to downregulate the activities of Caspase-3 and Caspase-9, inhibit the inflammatory levels of TNF-α, IL-6, IL-8IL-1β, and upregulate the expression of anti-apoptotic protein Bcl-2, which effectively inhibited endogenous apoptotic signaling, and alleviated the structural tissue destruction of colonic tissues and infiltration of inflammatory factors to a certain extent in UC mice (Zhu et al., 2021). The above findings suggest that all these compounds can modulate endogenous mitochondrial pathways, such as the anti-apoptotic protein Bcl-2, to achieve the treatment of UC, and such drugs may be candidates for the treatment of UC in the future.

#### 3.1.3 Compounds related to JAK/STAT signaling pathway

The JAK/STAT signaling pathway consists of the ligand-receptor complex, JAK and STAT. It is one of the critical nodes for maintaining normal cellular function. There is much evidence that aberrant expression of JAK/STAT is associated with many diseases. Inhibition of this signaling pathway is expected to be a new therapeutic tool. The JAK/STAT signaling pathway is a crucial regulator of apoptosis in UC cells, which plays a vital role in treating UC (Cordes et al., 2020). Daphnetin is a natural coumarin compound with outstanding efficacy in anti-inflammatory, antioxidant, and anti-apoptotic effects. Some scholars have shown by DSS-induced UC mice that daphnetin (16 mg/kg) was able to downregulate the expression of IFN-γ, IL-1β, IL-6, TNF-α, and upregulate the expression of ZO-2, occludin and claudin-1, and Bcl-2, which effectively alleviated the structural damage of intestinal mucosa. The mechanism of action was related to the downregulation of JAK2/STAT signaling expression to inhibit apoptosis. In addition, the *in vitro* experiment LPS-induced Caco-2 cell model confirmed this phenomenon. Since no positive control group was set up in this experiment, the superiority of daphnetin could not be confirmed (He et al., 2023). Grape-seed polyphenols (GSP) are natural compound extracted from grape seed with anti-inflammatory and anti-apoptotic pharmacological effects. In DSS-induced UC model mouse experiments, GSP (500,750 mg/kg) was used to treat UC mice by gavage without a positive control group. The results showed that GSP could reduce the levels of inflammatory cytokines IL-6, IL-1β, and TNF-α, alleviate the clinical symptoms of UC, downregulate the phosphorylation of STAT3, and inhibit the apoptosis of intestinal epithelial cells (Wang et al., 2020). Matrine is an alkaloid extracted from the dried roots, plants, and fruits of the leguminous plant *Sophora flavescens* Ait. It has a variety of pharmacological effects, including anti-inflammatory and anticancer effects. Chen et al. established *in vitro* experiments of DSS treatment of NCM460 cells and found that matrine was able to effectively reduce the inflammation in the NCM460 cell model factor IL-1β, IL-6, and TNF-α, IL-2 levels, inhibited the expression of Bax, Caspase-3, increased the expression of anti-apoptotic Bcl-2 protein, significantly inhibited apoptosis and inflammatory infiltration. The mechanism of action was related to the inhibition of JAK/STAT (Chen et al., 2022). In summary, these natural compounds may become new ways to treat UC by regulating the JAK/STAT signaling pathway.

#### 3.1.4 Compounds related to ERK signaling pathway

ERK is a regulatory kinase for extracellular signaling and contains two types of ERK2/ERK. Notably, ERK promotes the Caspase-8 signaling pathway to turn on the exogenous apoptosis pathway and regulates the Bcl-2 family of proteins to activate the endogenous apoptosis pathway. We have also found these several natural compounds mediating the ERK pathway to treat UC to be of great significance. Aloe is a concentrated dried substance of the sap of the leaves of *Aloe barbadensis* Miller., feroxmiller or other closely related plants of the same genus in the lily family. It has been shown to have anti-inflammatory, antimicrobial, and wound-healing properties (Sánchez et al., 2020). Shi *et al.* treated DSS-induced UC rats by gavage with different doses of Aloe extract (18.72 mg/kg) for 10 days 5-ASA (400 mg/kg) was used as a positive control group. The results showed that aloe could inhibit IL-6, IL-1β, and TNF-α levels to alleviate inflammatory infiltration and enhance colonic mucosal secretion and layer thickness. In addition, the LPS-induced human colonic cuprocyte LS174T *in vitro* model experiment also proved that aloe extract has good anti-inflammatory activity. However, this experiment has some shortcomings, such as failing to clarify which components in aloe extract play a therapeutic role (Shi et al., 2021). Ginsenoside RK2 is a dammarane triterpenoid saponin isolated from *Panax ginseng* CA Mey., in the family of Pentaphyllaceae, which has anti-inflammatory, antioxidant, and anti-apoptotic effects. Huang *et al.* used colon adenocarcinoma Caco cells and human intestinal epithelial cells THP-1 to establish UC *in vitro* modeling experiments. The present study was not set to conduct *in vivo* experiments, and there was no positive control group. The results of the *in vitro* study revealed that Ginsenoside RK2 (5,10,20,40 μM) effectively reduced IL-6, IL-1β, and TNF-α expression and acted in a concentration-dependent manner. In addition, the Western blot assay showed that the mechanism of action was achieved by down-regulating the ERK/MEK signaling pathway and inhibiting apoptosis (Huang et al., 2022). The above studies indicate that ERK is the target of natural compounds that alleviate UC by inhibiting apoptosis.

#### 3.1.5 Compounds related to PI3K/AKT signaling pathway

Phosphatidylinositol 3-kinase (PI3K) is a lipid kinase, and all three forms of the protein are involved in cellular signaling. AKT is thought to be an anti-apoptotic regulator that promotes cancer progression, and active AKT proteins can activate endogenous pathways of apoptosis to restore the anti-apoptotic function of Bcl-2 and Bcl-xl and also inhibit the activation of the caspase-9 protein AKT can also inhibit the activation of caspase-9 protein, which can hinder apoptosis (Li et al., 2018). As an essential signaling pathway, PI3K/AKT is crucial in regulating cell proliferation, apoptosis, and transformation. There is a strong correlation between the PI3K/AKT signaling pathway and the course of UC as an inflammatory disease. There is evidence that inhibition of the PI3K/AKT signaling pathway blocks apoptosis and thus significantly improves the clinical symptoms of UC (Liu et al., 2021). Therefore, the following compounds target the PI3K/AKT signaling pathway and have made new progress in improving the pathological status and clinical symptoms of UC. Chen et al. injected oxymatrine (OMT) (25,50,100 mg/kg) intraperitoneally in a DSS-induced UC model for 7 consecutive days without a positive control group. The results significantly reduced TNF-α, IL-6, IL-1β, IFN-γ, and IL-17A levels, effectively alleviating colonic inflammatory injury and tissue edema. The mechanism was achieved by inhibiting the PI3K/AKT signaling pathway. It was further found that the efficacy of 50 mg/kg OMT was better than 100 mg/kg, but the two were not statistically significant (Chen et al., 2017). Rhein is an anthraquinone extracted from the dried roots and rhizomes of the Polygonaceae plant *Rheum palmatum* L., *Rheum tanguticum* Maxim. Ex Balf. or *Rheum officinale* Baill., which plays an anti-inflammatory, anti-tumor, and antioxidant role in the treatment of diseases (Cheng et al., 2021). Dong *et al.* intervened in DSS-induced UC model mice with rhein (12.5,25,50 mg/kg) and S-ASP (200 mg/kg) as a positive control group. Rhein significantly improved the symptoms of UC mice, such as colon shortening, weight loss, diarrhea, and blood in the stool. Using Western blotting assay, we found that rhein inhibited the phosphorylated protein levels of PI3K/Akt/mTOR and p70S6K1 and improved intestinal dysbiosis by increasing beneficial bacteria and decreasing harmful bacteria. In addition, we found that the expression of IL-6, IL-1β, and TNF-α was inhibited, and the therapeutic effect of rhein (50 mg/kg) was comparable to that of S-ASP (Dong et al., 2022). Therefore, the PI3K/AKT signaling pathway is a potential target for natural products that inhibit UC by inducing apoptosis.

#### 3.1.6 Compounds targeting oxidative stress induced apoptotic

Oxidative stress is a state of stress due to an imbalance between producing oxides such as ROS and available antioxidant defenses. Numerous studies have demonstrated that oxidative stress can cause apoptosis through both mitochondria-dependent and mitochondria-independent pathways (Huang, et al., 2022). Improving UC by inhibiting apoptosis through regulating oxidative stress-related signaling pathways has been widely recognized (Li et al., 2023). In response to the above studies, we found several compounds that demonstrate effectiveness in modulating this pathway. Honey polyphenols, as a natural antioxidant, have been shown to have anti-inflammatory properties (Ranneh et al., 2021). Scutellarin is a flavonoid that has been tapped for its pharmacological effects, such as anti-apoptotic, antioxidant, and anti-inflammatory effects in treating UC. Aksit et al. investigated the mechanism of action of scutellarin (20 mg/kg) on colitis by AA-induced UC rats, with sulfasalazine (100 mg/kg) as a positive control group. The results showed that both scutellarin and sulfasalazine effectively alleviated the pathological damage of colonic mucosa and reduced the expression of inflammatory factors by inhibiting oxidative stress and apoptosis. In addition, IL-6, TNF-α, MDA, and Bax were significantly reduced, and SOD and Bcl-2 protein expression was upregulated (Aksit et al., 2023). Cardamonin is a natural chalcone derivative with excellent performance in anti-inflammatory, immunomodulatory, and anticancer properties. Ali et al. treated UC model rats by concentration gradient method using cardamonin (10,30 mg/kg) by gavage for 14 days. The colon was analyzed by histopathology and ELISA. The results revealed that cardamonin reduced the expression of MPO, iNOS, NF-κB, TNF-α, and MDA, and immunohistochemistry showed inhibition of caspase-3 expression in the treated group. This effect may alleviate colonic inflammation and pathological damage by inhibiting oxidative stress and apoptosis. However, no positive control group was set up in this experiment, and the reliability of the experimental findings should be further verified (Ali et al., 2017). Oleracein E (OE) is a phenolic alkaloid extracted from the alcoholic extract of *Portulaca oleracea* L., which has been shown to possess antioxidant properties. Huang *et al.* used OE to treat TNBS-induced UC rats without a positive control group. The results showed that OE (5,10,20 mg/kg) significantly attenuated the levels of IL-6, TNF-α, and IL-1β, downregulated the expression of Bax, Caspase-3, and MDA, and upregulated the content of Bcl-2, CAT, claudin-2, ZO-1, and occludin, which effectively mitigated oxidative stress damage, reduced colonic inflammation, and repaired the intestinal mucosal barrier, of which OE (20 mg/kg) was the most effective. *In vitro* experiments demonstrated that OE could also ameliorate LPS-induced colonic inflammatory injury in Caco-2 cells (Huang et al., 2023). The above studies show that oxidative stress-related signaling pathways are meaningful ways for natural products to treat UC.

### 3.2 Compounds that regulate the autophagy pathway

Autophagy regulation is increasingly becoming a new way to treat UC. Promoting or inhibiting key targets can alleviate UC, repair colon tissue, improve its pathological state, and enrich the number of beneficial bacteria in colon tissue. The following compounds will provide scientific evidence for this.

#### 3.2.1 Compounds related to AMPK/mTOR signaling pathway

The studies mentioned above have shown that AMPK/mTOR is a core link in the autophagy response and a highly potential target in the treatment of UC disease. Therefore, research on compounds to treat UC mostly focuses on this signaling pathway. Procyanidin A1(PCA1) are polyphenolic compounds isolated from proanthocyanidins and have various biological activities such as anti-inflammatory (Han et al., 2019). Zhang et *al*. intervened in DSS-induced UC mice with different doses of PCA1 (5.10 mg/kg) for 6 days without a positive control group. Western blot and ELISA were used to determine protein molecules and inflammatory expression. The results showed that PCA1 decreased IL-6, TNF-α, and IL-Iβ inflammatory factors levels, increased the ratio of Beclin-1 and LC3II/I, and downregulated the expression of P62 protein. Meanwhile, *in vitro* and *ex vivo* experiments further clarified that this mechanism of action was realized by inhibiting AMPK/mTOR (Zhang et al., 2022). Dioscin is a steroidal saponin isolated from a variety of vegetables and herbs. It has powerful anti-inflammatory, immunomodulatory, and hypolipidemic effects (Tao et al., 2018). Li *et al.* explored the protective mechanism of dioscin against colitis by DSS-induced UC mice. The results showed that dioscin (40 mg/kg) was able to promote AMPK phosphorylation, downregulate MDA, upregulate SOD and GSH levels, inhibit mTOR activation, and promote autophagy to alleviate colonic inflammation. The relationship between body weight and administered dose needed to be established in this study, which affected the reliability of the experimental data (Li et al., 2022). Phellodendrine, the main alkaloidal constituent of the dried bark of *Phellodendron chinense* Schneid or *Phellodendron amurense* Rupr. of the Brassicaceae family has been shown to have anti-inflammatory effects (Yang et al., 2017). Su et al. used a DSS-induced model mice experiments and found that both phellodendrine (30 mg/kg) and sulfasalazine group (40 mg/mL) were effective in alleviating colonic shortening, pathological damage index, mucosal inflammation, and repairing crypt structure. Western blot assay showed that phellodendrine upregulated AMPK/mTOR and LC3-II/LC3-I expression and promoted autophagy. The design of this experiment needed to be more rigorous; for example, the drug concentration gradient experiment was not performed (Su et al., 2021). It is worth noting that Zhang et al. used curcumin (50 mg/kg) and resveratrol (80 mg/kg), respectively, to intervene in the treatment of DSS-induced UC mice without a positive control group. The results showed that curcumin and resveratrol significantly ameliorated diarrhea and rectal bleeding, repaired crypt structures, and reduced inflammation levels in UC model mice. It also downregulated the levels of TNF-α and IL-6 inflammatory factors. It inhibited the expression of autophagy-related proteins Atg12, Beclin-1, and LC3II, revealing that its mechanism of action was related to SIRT1/mTOR signaling (Zhang et al., 2019). Chlorophyllin (CHL) is a water-soluble derivative of chlorophyll, which has been proven to have antioxidant, anti-cancer, and other properties (Solymosi and Mysliwa-Kurdziel,2017). Zhang *et al.* found that oral administration of CHL (40 mg/kg) reduced DSS-induced inflammation in the colon and structural alterations of intestinal epithelial cells in mice and, at the same time, reduced the degree of colonic shortening and clinical symptoms (such as diarrhea, blood in stool) and mRNA levels of TNF-α and IL-Iβ. In addition, we found that chloroquine, an autophagy inhibitor, exhibited the same effect as CHL (Zhang et al., 2022). Berberine (BBR) is a natural quaternary quinoline-based alkaloid with anti-inflammatory, antioxidant, anti-cancer, and other pharmacological properties (Lu et al., 2022). Xu et al. established a DSS-induced UC mouse model to clarify the mechanism of BBR’s action in treating colitis. The experiment was divided into 6 groups (control group, DSS group, DSS + NS group, BBR 25, 50, 100 mg/kg group), and no positive control group was set. The experimental results showed that BBR could promote the expression of p-AMPK, P-ULK1, LC3B, inhibit the secretion of TNF-α and IL-17A, enhance the content of IL-4 and IL-13, and improve the degree of damage such as histological score, villus length, crypt depth, etc. The therapeutic effect was dose-dependent with BBR, and the mechanism was related to regulating the AMPK/MTER/ULK1 pathway to promote autophagy (Xu et al., 2022). Luteolin is a natural flavonoid compound that has anti-inflammatory, anti-cancer, and other activities (Imran et al., 2019). Yuan *et al.* treated DSS-induced UC mice with luteolin (50 mg/kg) by gavage for 14 days, and sulfasalazine (300 mg/kg) was used as a positive control group. The colon’s pathological organization and inflammation expression were observed by HE staining, ELISA, and Western blot. The results showed that the luteolin group significantly downregulated IL-6, IL-17, and IL-23 inflammation levels and enhanced SIRT3 protein expression and phosphorylation levels of AMPK. In addition, we also observed a significant improvement in the depth and extent of colonic ulcers in UC mice compared with the previous ones and a decrease in the degree of inflammatory cell infiltration and pathohistological scores. This study still has things that could be improved, such as no concentration gradient setting and unclear clinical effects (Yuan et al., 2023). Alpinetin (ALPI) is a flavonoid compound derived from various edible and medicinal plants, and modern pharmacology has confirmed the anti-inflammatory, hemostatic, antioxidant, and anticancer properties of ALPI (Gul et al., 2022). Miao *et al.* demonstrated that ALPI (30 mg/kg) upregulated the expression of LC3B-II, Beclin −1, p62, Atg7, and Atg5 while improving intestinal barrier homeostasis and inhibiting apoptosis of monolayer columnar intestinal epithelial cells (IECs) to attenuate the disruption of the intestinal epithelial barrier and that its mechanism of action was related to the AhR/suv39h1/TSC2/mTORC1/autophagy pathways (Miao et al., 2019). These results show that regulating autophagy-related targets can effectively alleviate UC and improve the clinical symptoms and pathological status of UC, and may become a new way to treat UC in the future.

#### 3.2.2 Compounds related to Beclin-1 signaling pathway

Beclin-1 is a critical regulatory protein in the autophagy process and is responsible for autophagosome membrane formation. Especially in the treatment of UC, upregulation of the expression of this protein can effectively activate autophagy expression, reduce the inflammatory response, and improve the pathological state of the colonic mucosa to achieve the alleviation of UC. Therefore, the targeted regulation of Beclin-1 of these several compounds can reflect this effect. Resveratrol (Res) are natural polyphenolic compounds with anti-inflammatory, antioxidant, and antitumor properties extracted from grapes and wine (Zhang et al., 2021). Pan et al. used DSS to establish UC model mice, and 5-ASA (200 mg/kg) was used as a positive control group. After treatment by Res (100 mg/kg), inflammatory factors such as TNF-α, IL-1, and IL-42β were significantly downregulated in serum, and the expression of tight junction proteins occludin and ZO-1 was upregulated. In contrast, LC3B and Beclin-1 expression was increased. This suggests that alleviating inflammation and pathological tissue scores of intestinal mucosa in UC mice is associated with the enhancement of autophagy by resveratrol. In addition, the experiments also revealed that Res was superior to 5-ASA in protecting the integrity of the colonic barrier (Pan et al., 2020). Paeoniflorin is derived from the monoterpenoids in the roots of the Ranunculaceae plant *Paeonia lactiflora* Pall. It has anti-inflammatory, analgesic, anti-tumor, immunomodulatory, and other properties (Zhang et al., 2020; Zhang and Wei, 2020). Li *et al.* reported that the mechanism of action was explored by treating DSS-induced UC rats by gavage with paeoniflorin (2.5 g/kg) for 21 days, with sulfasalazine (0.5 g/kg) as a positive control group. The results showed that paeoniflorin promoted cellular autophagy and restored intestinal homeostasis by up-regulating the activity of Beclin-1 and inhibiting Bcl-2, IL-6, and TNF-α levels, effectively alleviating UC (Li et al., 2020). Therefore, Beclin-1 becomes a potential target of natural products to inhibit UC intestinal mucosal inflammation and improve the pathological state of UC by regulating autophagy.

#### 3.2.3 Compounds related to the NF-κB signaling pathway

In addition to apoptosis, NF-κB is also a very important target in the autophagy response and has been deeply studied in the regulation of autophagy in UC. Targeting the NF-κB signaling pathway to regulate autophagy has potential significance in the treatment of UC. Cao et al. showed that BBR downregulated the inflammatory levels of TNF-α, IL-6, and IL-β, improved UC pathology, promoted intestinal epithelial cell proliferation, maintained cellular metabolism, and restored intestinal barrier function. The mechanism was related to the inhibition of the TLR4/NF-κB signaling pathway by BBR (Cao et al., 2022). Baicalin (BA) can protect the intestinal mucosal barrier by improving autophagic flux and inducing apoptosis to control UC inflammation. RIZZO *et al.* treated LPS-induced HT-29 intestinal epithelial cell model with different concentrations of BA (1–10 μg/mL). The results showed that BA-treated HT-29 cells suppressed the expression of TNF-α and IL-1β inflammatory factors and upregulated the protein levels of Claudin 1 and LC3. Since this experiment was not validated *in vivo* and clinically, the drug’s effectiveness is difficult to guarantee (RIZZO et al., 2021). Hyperoside (Hyp) is a flavonol glycoside compound derived from the fruits and phytomedicines of plants in the family Hypericum, Rhododendron, and others. It has biological activities such as anti-inflammatory, anticancer, and antioxidant (Xu et al., 2022). YU *et al.* treated TNBS-induced UC rats by gavage with different concentrations of Hyp (25/50/100 mg/kg) for 7 days, and SASP (200 mg/kg) was used as a positive control group. The results showed that Hyp decreased inflammatory expression, increased SOD activity, and showed synergistic effects with SASP by modulating IL-1β, TNF-α, MDA, and MPO levels with comparable therapeutic effects. They also found that Hyp simultaneously reduced LC3-II, Beclin1, and p62 protein expression and regulated autophagic flow to alleviate colonic mucosal edema and necrosis. The NF-κB pathway was revealed to be the signaling mechanism for the anti-inflammatory effect of Hyp (Yu et al., 2021). In addition, evodiamine (EVO) is a quinolone-like organism derived from the plant *Evodia rutaecarpa* (Juss.) Benth. In the family Rutaceae, which has been found to have anticancer and anti-inflammatory potential (Panda et al., 2023). EVO (20.40,60 mg/kg) and 5-ASA (100 mg/kg) positive control groups were both able to ameliorate colonic inflammation in UC model mice. Mechanistically, EVO was able to mediate the activation of the autophagy pathway by NF-κB inhibiting the activity of NLRP3 inflammatory vesicles to downregulate the levels of inflammatory factors (IL-1β, IL-18) and upregulate the expression of LC3-II. In addition, the experiment further administered EVO (60 mg/kg) to UC mice, and it was found that there was no statistically significant difference in body weight change and colon length (DING et al., 2020). Table 2 lists other natural compounds that alleviate UC by modulating autophagy induced by NF-κB-related signaling pathways. The above studies suggest that modulation of NF-κB signaling by these compounds is considered a vital alternative to improve UC’s clinical symptoms and pathology.

**TABLE 2:** Natural compounds target autophagy signaling pathway to treat UC.

Natural products	Dosage	Models	Biological effects	Results	Mechanism	References
Evodiamine	20.40,60 mg/kg	UC model rats	MPO↓IL-1β↓1L-18↓ASC↓Caspase-1↓LC3-II↑LC3-I↑	Modulation of autophagy to inhibit inflammatory infiltration and alleviate colonic pathologic damage	NF-κB p65↓	DING et al. (2020)
	10 μM	THP-1 cells				
Ginsenoside Rd	10.20,40 mg/kg	UC model rats	IL-1β↓ IL-6↓Caspase-1↓TNF-α↓GSH↑	Inhibit the expression of inflammation and alleviate UC	NLRP3↓	Liu et al. (2018)
Salidroside	15 mg/kg	UC model rats	IL-6↓IL-10↓TNF-α↓NLRP3↓ASC↓Caspase-1↓	Inhibition of autophagy, alleviation of colonic edema, repair of colonic structure	NF-κB↓	Liu et al. (2019)
paeoniflorin	2.5 mg/kg	UC model rats	TNF-α↓IL-6↓Beclin-1↑Bcl-2↓	Inhibition of inflammatory response and amelioration of pathological damage to the colonic mucosa	Beclin-1↑	LI et al. (2020)
Potentilla discolor Bunge	0.18,0.36,0.72 g/kg	UC model rats	NF-κB↑P62↑parkin mRNA↑IL-6↓IL-10↑	Promoting mitochondrial autophagy, altering inflammatory activity, and alleviating colonic mucosal damage	NF-κB↑	Liu et al. (2021)
Lonicerin	3,10,30 mg/kg	UC model rats	IL-1β↓IL-18↓NLRP3↓ASC↓Caspase-1↓	Inhibits activation of NLRP3 inflammatory vesicles and significantly reduces colonic inflammation	NLRP3↓	Lv et al. (2021)
	1,3,10,30 μmol/L	THP-1 cells				
Dioscin	40 mg/kg	UC model rats	MDA↓SOD↑GSH↑	Promote autophagy and play an anti-inflammatory and anti-oxidative stress role	mTOR↓	Li et al. (2022)
Alpinetin	30 mg/kg	UC model rats	LC3 B -II↑Beclin −1↑p62↑Atg7↑Atg5↑	Improving intestinal barrier homeostasis and inhibiting apoptosis of monolayer columnar intestinal epithelial cells (IECs) to attenuate intestinal epithelial barrier disruption	mTORC1↓	Miao et al. (2019)
	10,30 μM	Caco-2/NCM460 cells				
palmatine	10,40,100 mg/kg	UC model rats	MPO↓IL-1β↓TNF-α↓PINK1↑Parkin↑	Inhibits inflammatory damage and repairs damaged colonic mucosa	NLRP3↓	Mai et al. (2019)
Resveratrol	100 mg/kg	UC model rats	TNF-α↓IL-1↓IL-42β↓occludin↑ZO-1↑LC3B↑Beclin-1↑	Inhibition of inflammatory expression and amelioration of pathological damage to the colonic mucosa	Beclin-1↑	PAN et al. (2020)
Baicalin	1–10 μg/mL	HT-29 cells	TNF-α↓IL-1β↓IL-6↓Claudin 1↑mRNA↓	Inhibits inflammatory response and improves paracellular permeability	NF-κB↓	RIZZO et al. (2021)
Phellodendrine	30 mg/kg	UC model rats	LC3-II/LC3-I↑TNF-α↓	increased the abundance of intestinal flora and the number of beneficial bacteria, and significantly improved the colon pathology and pathological damage index of mice induced by DSS	AMPK/mTOR↑	Su et al. (2021)
M10	50–100 mg/kg	UC model rats	IL-6↓TNF-α↓CD8+T↑CD4+T↑	Inhibits autophagic response and reduces inflammatory infiltration	NF-κB/IL-6/STAT3↓	Wang et al. (2018)
Berberine	25,50,100 mg/kg	UC model rats/	p-AMPK↑P-ULK1↑LC3B↑TNF-α↓IL-17A↓	Inhibited inflammatory secretion and improved histologic scores, chorionic villus length, crypt depth, and other degrees of injury	AMPK/mTOR↑	Xu et al. (2022)
	100 μM	ICE-18 cells				
Hyperoside	25,50,100 mg/kg	UC model rats	IL-1β↓TNF-α↓MDA↓LC3-II↓Beclin 1↓p62↓	Modulation of autophagic flow to alleviate colonic mucosal edema and necrosis	NF-κB p65↓	Yu et al. (2021)
luteolin	50 mg/kg	UC model rats	IL-6↓IL-17↓IL-23↓mRNA↓IL-6↓IL-17↓IL-23↓	Improvement of pathologies such as edema, erosion and ulceration of colonic tissues	AMPK/mTOR↑	Yuan et al. (2023)
Naringin	50,100 mg/kg	UC model rats	GM-CSF↓M-CSF↓IL-6↓IL-10↓TNF-α↓	Inhibition of autophagic response and mitigation of pathological damage	NF-κB↓	Zhang et al. (2018)
Curcumin/Resveratrol	50/80 mg/kg	UC model rats	TNF-α↓IL-6↓Atg12↓Beclin-1↓LC3II↓	Inhibits intestinal inflammatory cascade and protects colonic barrier function	SIRT1/mTOR↑	Zhang et al. (2019)
Chlorophyllin	40 mg/kg	UC model rats	TNF-α↓IL-1β↓mRNA↓LC3II↓P62↑	Inhibit inflammatory damage and repair intestinal mucosal barrier	Akt/mTOR↑	Zhang et al. (2022)
Procyanidins A1	5.10 mg/kg	UC model rats	P62↑P-AMPK↑Beclin-3↑p-mTOR↓	promote cell autophagy and reduce the expression of pro-inflammatory factors, and alleviate the pathological state of UC	AMPK/mTOR↑	Zhang et al. (2022)
	40,80 μM	HT-29cells/IEC-6 cells				

#### 3.2.4 Compounds related to NLRP3 signaling pathway

Numerous studies have shown that NLRP3 can be degraded through the autophagy pathway and inhibit NLRP3 inflammasome activity to antagonize the damage of inflammation to host cells, thus exerting a protective effect. Fortunately, several compounds have emerged as drug candidates for treating UC. Lonicerin is a flavonoid glycoside from the vine stem of Honeysuckle, *Lonicera japonica*, family Loniceraceae, with pharmacological effects such as anti-inflammatory and immunomodulatory effects (Yang et al., 2023). Lv et al. treated DSS-induced mice with different doses of lonicerin (3,10,30 mg/kg) by gavage for 10 days.5-ASA (200 mg/kg) was used as a positive control group. The results showed that lonicerin reduced the pathological signs of DSS-induced UC, promoted the degradation of NLRP3 lysosomes, enhanced ATG5-mediated autophagy, and inhibited the expression of inflammatory factors IL-18 and IL-1β. The lowest effective dose of lonicerin was 10 mg/kg in a dose-dependent manner. This may affect cellular autophagy by regulating the EZH2/ATG5/NLRP3 signaling pathway (Lv et al., 2021). Palmatine is an isoquinoline alkaloid with anti-inflammatory and antimicrobial activities. Mai et al. found in experiments on the treatment of UC mice that both palmatin (10,40,100 mg/kg) and the sulfasalazine pyridine group (200 mg/kg) were able to inhibit the activation of the NLRP3 inflammatory vesicles significantly and the levels of IL-1β, TNF-α, and F4/80+ cell counts, while the mitochondrial autophagy inhibitor cyclosporine A (CsA) was found to attenuate the therapeutic effect of palmatin on UC. This suggests that the therapeutic mechanism of palmatin is closely related to mitochondrial autophagy. However, this experiment was not administered according to body weight, and the conclusion was not rigorous enough (Mai et al., 2019). Table 2 lists other natural compounds that alleviate UC by modulating autophagy induced by the NLRP3-related signaling pathway. The above compounds suggest that targeting the regulation of NLRP3-related signaling pathways provides new perspectives and therapeutic strategies for treating UC.

### 3.3 Compounds that regulate cellular ferroptosis pathway

Much scientific evidence has proved that Ferroptosis can effectively treat UC, which can effectively improve the clinical symptoms, pathological state, and disease activity index of UC and reshape the intestinal mucosal barrier. The following compounds have demonstrated the therapeutic effects of UC by targeting critical signaling pathways related to ferroptosis.

#### 3.3.1 Compounds related to Nrf2 signaling pathway

Numerous studies have demonstrated that Nrf2 is involved in the expression of multiple transcription factors in the body and plays a vital role in various diseases. When stimulated by external stimuli (e.g., TNF-α, ROS), Nrf2 dissociates from Keap-1 and initiates the expression of downstream target genes using nuclear transcription to protect cells from damage. Chen et al. found that activation of the Nrf2 signaling pathway positively ameliorated UC through experiments establishing a UC model. Thus, several of these natural compounds showed anti-UC effects by modulating Nrf2 signaling pathway-mediated ferroptosis (Chen et al., 2020). Astragalus polysaccharide (APS) is a macromolecular polysaccharide extract of *Astragalus membranaceus* (Fisch.) Bge. From the legume family of Astragalus mongolia with anti-inflammatory, antioxidant, anticancer, and immunomodulatory effects (Li et al., 2022). APS (100,200,300 mg/kg) inhibited ferroptosis, downregulated the expression of ferroptosis-related genes (PTGS2, FTH, and FTL), improved colon length and disease activity index, and effectively mitigated UC disease progression in UC model mice, and its therapeutic mechanism was related to the Nrf2/HO-2 pathway; however, a positive control group was not set up in the present experiment, and the conclusions obtained were insufficient (Chen et al., 2021). It is reported that codonopsis is the dry root of *Codonopsis pilosula* (Franch.) Nannf., a plant of the Campanulaceae family, which has anti-inflammatory, antioxidant, anti-tumour, immunomodulatory, neuroprotective, and other properties (Dong et al., 2023). Li *et al.* intervened in TNBS-induced UC rats with codonopsis extract (4.5,9,18 g/kg) for 7 days. SASP (0.3 g/kg) was used as a positive control. HE staining was used to analyze the pathology of the colonic mucosa for tissue damage and repair of the colonic mucosal barrier. ELISA showed a significant decrease in IL-1β, IL-6, IL-8, and Fe2+, and the biochemical assay detected upregulation of SOD, GSH, and GPX4 content, and MDA expression was suppressed. Mechanistic studies showed that codonopsis extract could regulate the Keap1/Nrf2 signaling pathway to inhibit ferroptosis (Li et al., 2023; Fang et al., 2023). These experimental results mean that modulating Nrf2 can be used as a natural target for natural products to treat UC.

#### 3.3.2 Compounds related to GPX4 signaling pathway

GPX4 protein is a significant player in ferroptosis and can function as a disease target and play a therapeutic role in treating diseases. An animal experiment showed that the activation of the GPX4 protein pathway could significantly inhibit ferroptosis and exert a protective effect on intestinal mucosal structures (Li et al., 2022). Thus, we identified the following therapeutic effects of several natural compounds realized through GPX4 targeting. Protocatechuic Acid (PCA) is a natural phenolic acid primarily found in plant medicines and diets with potent anti-inflammatory, anticancer, and anticarcinogenic properties (Song et al., 2020). Yang *et al.* intervened in DSS-induced UC mice for 7 days using PCA (5,10,20 mg/kg). SASP (100 mg/kg) was used as a positive control group. The results showed that the expression levels of IL-6, IL-12, TNF-α, and MDA were significantly decreased, and the activities of GSH and GPX4 were enhanced in colon tissues by PCA treatment. The pathological state of colon tissue and ulcer area was significantly improved. Meanwhile, the 16SrDNA results showed that the relative abundance of *mycobacterium* anisopliae phylum and warty microbiota was significantly increased compared with the model group. In addition, the inhibition of ferroptosis was similarly demonstrated to alleviate this phenomenon of UC in the experiment of ferroptosis agonist Erastin treatment of Caco-2 cells (Yang et al., 2023). In addition, Curculigoside (CUR), a natural phenolic glycoside compound present in *Curculigo orchioides* Gaertn, possesses potent anti-inflammatory, antioxidant, and other biological activities (Liu et al., 2021). Wang et al. CUR (50,100 mg/kg) intervened in DSS-induced UC mice without a positive control group. CUR was able to ameliorate colonic mucosal erosions and colon shortening. In addition, CUR was able to reverse the expression levels of MDA, ROS, SOD, GSH, and GPX4 in the model group. Fer-1 was also protective against histological lesions in UC mice. However, the effect of CUR on the intestinal flora was not investigated in this experiment, which needs to be clarified by further studies. These results confirm the potential role of natural product-induced Ferroptosis in treating UC, and more studies are needed to support this idea (Wang et al., 2020). These results confirm the potential role of natural products in inducing ferroptosis in the treatment of UC, and more studies are needed to support this view.

### 3.4 Compounds targeted to regulate the pyroptosis pathway

NLRP3 and GSDMD are recognized as critical regulatory proteins during pyroptosis response, and modulation of this target has far-reaching implications in treating UC and developing related inhibitors. Excitingly, we found several substances in some natural compounds that can induce GSDMD/NLRP3 to alleviate UC. Hydroxysafflor yellow A (HSYA) is a monochalcone glycoside extracted from the plant drug safflower, which has significant pharmacological effects such as anti-inflammatory and anticancer effects (Zhao et al., 2020). Chen *et al.* carried out a study to clarify the mechanism of HSYA on colitis. The DSS-induced UC mice were treated with HSYA (30.60 mg/kg) by giving DSS and 5-ASA (100 mg/kg) as a positive control group. The results showed that HSYA inhibited colonic inflammation and repaired colonic mucosal barrier function. The ability of HSYA to reduce the expression of inflammatory factors such as IL-1β, IL-6, TNF-α, and IL-18 by modulating the GSDMD/NLRP3 signaling pathway was confirmed by *in vivo* and *in vitro* studies. In addition, 16SrRNA sequencing showed that HSYA could also improve the gut microbial population in the model group (Chen et al., 2023). Honokiol is the main natural active product extracted from *Magnolia officinalis* Rehd. et Wils., which plays an important anti-inflammatory role in the treatment of UC. Wang et al. intervened in DSS-induced UC mice with honokiol (2.5.5 mg/kg) with mesalazine (66.7 mg/kg) as a positive control group. The researchers found that honokiol significantly improved colonic shortening, pathologic tissue score, and crypt structure, and enhanced intestinal barrier function. It also reduced TNF-α, IL6, IL-1β, and IFN-γ, mRNA levels, and inhibited GSDMD proteins to suppress cellular death. In addition, the experiment also showed that the honokiol (5 mg/kg) group had a better therapeutic effect than the mesalazine group (Wang et al., 2022). In addition, Forsythia suspensa Extract is a polyphenol-rich substance extracted from *Forsythia suspensa* (Thunb.) Vahl of the family Caryophyllaceae possesses anti-inflammatory, antioxidant, and antiallergenic effects. Chao *et al.* used Forsythia suspensa Extract (20, 40,80 mg/kg) to intervene in DSS-induced UC mice without a positive control group. The results showed that Forsythia suspensa Extract significantly reversed UC mice’s intestinal villi degeneration, necrosis, proliferation, and inflammatory infiltration. In addition, qRT-PCT showed that Forsythia suspensa Extract upregulated the protein activity of Nrf2 and enhanced the antioxidant capacity. Western blot inhibited the expression of ASC, Caspase-1, IL-18, and IL-1β. The same was demonstrated *in vitro* by the J774A.1 cell model (Chao et al., 2022). Table 3 lists other compounds that induce cellular pyroptosis to alleviate UC. The above results confirm that natural compounds targeting the regulation of the pyroptosis signaling pathway may be an effective treatment option for UC.

**TABLE 3:** Natural compounds target other signaling pathways to treat UC.

Natural products	Dosage	Models	Biological effects	Results	RCD types	Mechanism	References
Astragalus polysaccharide	100,200,300 mg/kg	UC model rats	MDA↓PTGS2↓FTH↓FTL↓GSH↑IFN-γ↓IL-6↓TNF-α↓IL-1β↓	Improve colon length and disease activity index, and effectively alleviate the development of UC	Ferroptosis	Nrf2/HO-2↓	Chen et al. (2021)
	50 μg/mL	Caco-2 cells					
Forsythia suspensa Extract	20.40,80 mg/kg	UC model rats	GSDMD↓IL-1β↓MDA↓SOD↑Caspase-1↓IL-18↓	Significantly reduce inflammatory damage and alleviate metabolic dysfunction	Pyroptosis	NLRP3↓	Chao et al. (2022)
Betaine	600 mg/kg	ASUC model rats	MDA↓MPO↓NOS ↓COX2↓GSH↑Nrf2↑CAT ↑SOD1↑NLRP3↓ASC ↓caspase-1↓	Inhibition of inflammatory expression and reversal of colonic shortening and colonic mucosal disorders	Pyroptosis	NLRP3/GSDMD↓	Chen et al. (2022)
Hydroxysafflor yellow A	30,60 mg/kg	UC model rats	IL-1β↓IL-6↓TNF-α↓IL-18↓	Inhibits pyroptosis pathway, decreases inflammatory response, increases gut flora abundance, and alleviates UC	Pyroptosis	NLRP3/GSDMD↓	Chen et al. (2023)
Portulaca	6,12,24 g/kg	UC model rats	NLRP3↓IL-1β,↓TNF-α,↓Il-1β,↓IL-6,↓IL-8,↓IL-17↓INF-γ↓	Inhibition of inflammatory expression and repair of intestinal barrier dysfunction	Pyroptosis	NLRP3↓	Fan et al. (2023)
Dihydrotanshinoe I	10,25 mg/kg	UC model rats	TNF-α↓IL-1β↓IL-6↓RIPK1↓RIPK3↓MPO↓	Reduces Disease Activity Index scores (DAI), improves pathology, and effectively exerts colonic mucosal protective effects	Necroptosis	RIPs-MLKL-caspase-8↓	Guo et al. (2018)
	0.1–0.5 μM	RAW264.7 cells					
Honeysuckle	40,80,120 mg/kg	UC model rats	LDH↓MDA↓TNF-α↓IL-6↓NLRP3↓ASC↓caspase-1↓IL-1β↓ mRNA↓SOD↑	Inhibits inflammatory infiltration and relieves intestinal mucosal damage	Pyroptosis	NLRP3/ASC/caspase-1↓	Ge et al. (2022)
celastrol	1 mg/kg	UC model rats	IL-1β↓IL-6↓MPO↓Caspase-8↑	Improvement of intestinal inflammation and pathological damage	Necroptosis	RIP3/MLKL↓	Jia et al. (2015)
Glycyrrhizae Radix	16 g/kg	UC model rats	MDA↓Fe2+↓IL-1β↓IL-6↓TNF-α↓GSH-P x↑FTH1↑SOD↑	Inhibited ferroptosis and reduced inflammatory infiltration	Ferroptosis	Nrf2/HO-2↑	Kong et al. (2023)
Ginsenoside Rg3	10 mg/kg	UC model rats	NLRP3↓GSDMD↓Caspase-1↓	Regulates intestinal flora homeostasis and resolves colon inflammation	Pyroptosis	NLRP3↓	Liu et al. (2023)
Codonopsis	4.5,9,18 mg/kg	UC model rats	Fe2+↓IL-1β↓IL-6↓IL-8↓TNF-α↓MDA↓ROS↓GSH↑SOD↑GPX4↑	Reduce ferroptosis of intestinal mucosal cells and restore the protective effect of intestinal mucosal barrier	Ferroptosis	Nrf2↑	Li et al. (2023)
Patchouli	10,20,40 mg/kg	UC model rats	TNF-α↓IFN-γ↓IL-1β↓IL-6↓IL-4↓IL-10↓ZO-1↑ZO-2↑claudin-1 ↑occludin↑	Inhibition the inflammatory response, relieves UC and maintains the integrity of the intestinal epithelial barrier	Necroptosis	RIP3/MLKL↓	Qu et al. (2017)
Cardamonin	20,40,80 mg/kg	UC model rats	RIPK1↓RIPK3↓TNF-α↓IL-1β↓IL-6↓	Inhibition of necrotic apoptosis and attenuation of intestinal barrier damage	Necroptosis	RIPK1/RIPK3↓	Shen et al. (2023)
Curculigoside	50,100 mg/kg	UC model rats	MDA↓ROS↓GSH↑SOD↑GPX4↑	Inhibition of Disease Activity Index, Histological Damage and Cell Death in Mice with Colitis	Ferroptosis	GPX4↑	Wang et al. (2020)
	100/200 μg/mL	ICE-6 cells					
Honokiol	2.5,5 mg/kg	UC model rats	IL-1β↓IL-6↓TNF-α↓IFN-γ↓GSDMD↓	Reducing inflammatory signals and restoring the integrity of the colon significantly reduces the severity of UC.	Pyroptosis	TLR4-NF-κB↓	Wang et al. (2022)
	5,10 μmol/L	RAW264.7 Cells					
*Periplaneta americana* extracts	0.05.0.1.0.2.0.4 mg/mL	RAW264.7 Cells	NF-κB p65↓NLRP3↓Caspase-1↓RIPK1↓RIPK3 ↓MLKL↓TNF-α↓IL-6↓IL-18↓IL-1β↓ZO-1↑Occludin↑Claudin-3↑	**i**nhibit inflammatory response and improve clinical symptoms and pathology	Necroptosis	RIP3/MLKL↓	Wu et al. (2023)
Rauwolfia ameliorate	100 mg/kg	UC model rats	NLRP3↓miR-124-3p↓TNFα↓IL-6↓IL-18↓IL-1β↓	Inhibition of NLRP3-mediated cellular pyroptosis attenuates colonic epithelial cell pyroptosis and mucosal damage in UC	Pyroptosis	NLRP3↓	Yuan et al. (2023)
	640 μg/mL	YAMC cells					
Protocatechuic Acid	5,10,20 mg/kg	UC model rats	IL-6↓IL-12↓TNF-α↓MDA↓GSH↑GPX4↑	To restore intestinal microbial homeostasis and reduce colonic inflammatory damage in mice by inhibiting ferroptosis	Ferroptosis	GPX4↑	Yang et al. (2023)
	20–80 μM	Caco-2 cells					
Hesperetin	20 mg/kg	UC model rats	TNF-α↓IL-1β↓IL-18↓HMGB1↓IL-6↓	Blocking activation of RIPK3/MLKL necro-apoptotic signaling to repair intestinal epithelial barrier function	Necroptosis	RIPK3/MLKL↓	Zhang et al. (2020)
	100 μM	Caco2/RAW264.7 cells					
Diacetylrhein	50,100 mg/kg	UC model rats	NLRP3↓IL-Iβ↓IL-6↓TGF-β↓ZO-1↑	Inhibits inflammatory response and reduces colon weight to length ratio, disease activity index and macroscopic damage	Pyroptosis	NF-κB↓	Zohny et al. (2022)

### 3.5 Compounds targeting the regulation of necrotizing apoptotic pathways in cells

Recent studies have demonstrated that some compounds can modulate RIPK1/RIPK3/MLKL-related signaling target protein molecules to inhibit necrotic apoptosis and alleviate UC. Hesperetin is a flavonoid compound widely found in citrus fruits with anti-inflammatory, antioxidant, and antitumor pharmacological effects (Muhammad et al., 2019). A study demonstrated the positive significance of Hesperetin (20 mg/kg) in alleviating colon inflammation, colon shortening, and repairing mucosal barrier function. Interestingly, Hesperetin could also significantly inhibit the expression of TNF-α, IL-1β, IL-18, HMGB1, and IL-6 by blocking the activation of RIPK3/MLKL necrotic apoptotic signaling. The study results would be more convincing if a concentration gradient and a positive control group could be established (Zhang et al., 2020). Celastrol is a triterpenoid isolated from the plant drug *Tripterygium wilfordii* Hook. f., which has been found to possess anti-inflammatory and anticancer activities (Chen et al., 2020). Treatment with celastrol (1 mg/kg) reduced IL-6, IL-Iβ, and TNF-α inflammation levels in DSS-induced UC mice, increased E-calmodulin and Caspase-8 levels, and improved colon shortening, and inflammatory infiltration of neutrophils. The mechanism of action may be related to the inhibition of the RIP3/MLKL necrotic apoptotic pathway by celastrol. The findings may be more adequate if a concentration gradient and a positive control group can be established (Jia et al., 2015). Cardamonin is a natural flavonoid derived from the ginger plant that plays a role in the treatment of inflammation, cancer, and immunomodulation (Wang et al., 2018). Wang et al. intervened DSS-induced UC mice with cardamonin (20,40,80 mg/kg) for 7 days without a positive control group. Cardamonin was able to improve colonic mucosal edema, weight loss, splenic index, and mucosal barrier. Cardamonin also inhibited IL-1, IL-6, MPO, and TNF-α inflammation levels. In addition, we could find the therapeutic mechanism related to phosphorylation of RIPK1/RIPK3/MLKL and thus inhibition of necrotic apoptosis by *in vitro* cellular HT-29 models. Cardamonin is a natural flavonoid derived from plants in the Zingiberaceae family and plays a role in the treatment of inflammation, cancer, immune regulation, *etc.* (Wang et al., 2018). Shen et al. used cardamonin (20,40,80 mg/kg) to intervene in DSS-induced UC mice for 7 days without setting up a positive control group. Cardamonin can improve colon mucosal edema, weight loss, spleen index, and mucosal barrier. Cardamonin also inhibited IL-1, IL-6, MPO, and TNF-α inflammatory levels. In addition, we can also find through the *in vitro* cell HT-29 model that its therapeutic mechanism is related to the phosphorylation of RIPK1/RIPK3/MLKL, thereby inhibiting necroptosis (Shen et al., 2023). Dihydrotanshinone I (DHT), one of the fat-soluble components extracted from *S. miltiorrhiza* Bge. In the family Labiatae, has pharmacological properties such as anti-inflammatory, antioxidant, and antitumor properties (Han et al., 2022). It has been reported that DHT (10.25 mg/kg) can inhibit the release of HMGB1, TNF-α, IL-1β, and IL-6 inflammatory factors, reduce the expression of RIP1, RIP3, MLKL, and significantly improve the pathological state, the degree of shortening of the colon, and the mucosal edema and erosion, and effectively exert the protective effect of colonic mucosa. *In vitro* experiments further revealed that the mechanism of action of DHT was related to the inhibition of the RIP1/RIP3/MLKL signaling pathway through the HT-29 cell model (Guo et al., 2018). Table 3 shows other compounds inducing necrotic apoptosis to alleviate UC. These results show the great potential of natural products in inducing necrotic apoptosis pathways to ameliorate UC, which should be investigated more in-depth.

## 4 Conclusion

UC is a chronic inflammatory response of the intestinal tract caused by the interaction between genetics and the external environment. Although its pathogenic mechanism is unclear, it is mainly related to defective barrier function, immune response, and intestinal microecological dysregulation. In recent years, with the rapid development of the economy, people’s dietary structure and living environment have led to a significant increase in the prevalence of UC. Although medical treatment has been revolutionized and various biologics and small molecule drugs have been developed, surgical treatment and postoperative complications have led to the deterioration of the quality of life of some patients.

Many scholars have found that PCD plays an essential role in the occurrence and development of UC. Regulating different forms of PCD can significantly improve the pathology of UC and restore the damaged colon. Therefore, in this paper, we reviewed the roles of different forms of PCD and critical signaling pathways in UC disease. Meanwhile, we identified apoptosis and autophagy as the primary forms of PCD and play an essential role in treating UC. In addition, we also observed that other forms of programmed death, such as cellular iron death and necrotic apoptosis, are less studied and should be further explored to provide a new basis for developing more compounds. According to reports, there are also some natural products, such as resveratrol, baicalin, berberine, etc., which can act on various forms of PCD and exert a colon protective effect. This may be related to the structure of the compound itself. It is worth noting that different forms of PCD also have some signals that interfere with each other, significantly enriching the therapeutic pathways of natural compounds and improving the therapeutic effect of drugs. Liu et al. found that Apple polyphenol extract could inhibit both apoptosis and pyroptosis pathways and Caspase-1/11. NLRP3, ASC, and increased Bcl-2 expression, effectively reducing inflammation levels and improving intestinal barrier integrity (Liu et al., 2021). Another study found that Luteolin activated the ERK signaling pathways anti-inflammatory, anti-apoptotic, and anti-autophagic activities, ameliorated colonic tissue damage, and suppressed inflammatory responses (Vukelić et al., 2020). This suggests that targeting signaling crosstalk may be a novel strategy for treating UC.

In the future, we should explore more compounds such as these to expand the choice of therapeutic agents for UC. Although these compounds are potential candidates for intervening in the course of UC by modulating PCD, most of them are basic studies with *in vivo* and *ex vivo* experiments, and there is a lack of high-quality clinical studies to determine the clinical effects of natural compounds. In addition, it is easy to find that these experimental designs and results also need more rigor. For example, there is a lack of positive control groups, no concentration gradient and comparison with the therapeutic effect of first-line drugs, and unclear toxic side effects and long-term effects. In addition, these natural compounds face some challenges, such as ambiguous pharmacological mechanisms and poor pharmacokinetics. Therefore, more research should be focused on clinically relevant clinical trials and long-term efficacy so that more compounds will emerge in the future to increase the diversity and selectivity of therapeutic agents and enhance the clinical efficacy of UC.

## Figures and Tables

**Table:** 

**UC**	Ulcerative colitis
**PCD**	Programmed cell death
**DD**	death domain
**MDA**	Malondialdehyde
**SOD**	Super oxide dismutase
**ERK**	extracellular signal-regulated kinase
**TAS**	total antioxidant status
**NO**	nitric oxide
**IL-6**	interleukin 6
**TNF-α**	tumor necrosis factor-α
**NLRP3**	NLR pyrin domain-containing 3
**NF-κB**	Nuclear factor kappa-B
**Bcl-2**	B-cell lymphoma-2
**Bax**	Bcl2-Associated X
**IL-4**	interleukin-4
**GSH**	Glutathione
**IL-β**	Interleukin-1β
**PI3K**	Phosphatidylinositol 3-kinase
**Akt**	Protein Kinase B
**mTOR**	mammalian target of rapamycin
**CAT**	catalase
**MPO**	Myeloperoxidase
**IFN-γ**	interferon gamma
**TGF-β1**	Transforming Growth Factor-β1
**GSH-Px**	Glutathione peroxidase
**IL-12**	interleukin 6
**IL-17**	interleukin 17
**NLRP3**	NLR family pyrin domain-containing protein 3
**IL-18**	interleukin 18
**MLKL**	Mixed lineage kinase domain-like protein
**RIPK1**	receptor-interacting protein kinase 1
**RIPK3**	receptor-interacting protein kinase 3
**GSDMD**	Gasdermin D
**ROS**	reactive oxygen species
**FTH1**	ferritin heavy chain 1
**GPX4**	Glutathione peroxidase 4
**NOS**	NO synthase
**INOS**	Inducible nitric oxide synthase
**Nrf2**	NFE2-related factor 2
**FTL**	ferritin light chain
**PTGS2**	Prostaglandin Endoperoxide Synthase 2
**ASC**	caspase activation and recruitment domain
**AMPK**	AMP-activated protein kinase
**LC3II**	light chain 3 II
**LC3I**	light chain 3 I
**p-ULk1**	phosphorylated ULK1
**Atg 12**	autophagy related 12
**FA**	Ferulic acid
**COP**	Coptisine
**Ori**	oridonin
**SA**	Sinapic Acid
**TAN**	Tanshinol
**OMT**	Oxymatrine
**OE**	Oleracein E
**PCA1**	Procyanidin A1
**BBR**	Berberine
**ALPI**	Alpinetin
**Res**	Resveratrol
**BA**	Baicalin
**Hyp**	Hyperoside
**EVO**	Evodiamine
**APS**	Astragalus polysaccharide
**PCA**	Protocatechuic Acid
